# Polymorphism of *BoLA-DRB3* in Semen and Its Influence on Progeny Derived from Semen with Resistance and Susceptibility to Bovine Leukemia Virus Proviral Load

**DOI:** 10.3390/pathogens14090837

**Published:** 2025-08-22

**Authors:** Aronggaowa Bao, Sonoko Watanuki, Ryosuke Matsuura, Yasunobu Matsumoto, Jinliang Wang, Hiroyuki Shimizu, Ayuha Niwano, Ryusaku Kawata, Yoko Aida

**Affiliations:** 1 Laboratory of Global Infectious Diseases Control Science, Graduate School of Agricultural and Life Sciences, 1-1-1 Yayoi, Bunkyo-ku, Tokyo 113-8657, Japan; bao-aronggaowa607@g.ecc.u-tokyo.ac.jp (A.B.); watanuki-sonoko228@g.ecc.u-tokyo.ac.jp (S.W.); matsuura-ryosuke@g.ecc.u-tokyo.ac.jp (R.M.); aymat@g.ecc.u-tokyo.ac.jp (Y.M.); 2 Laboratory of Global Animal Resource Science, Graduate School of Agricultural and Life Sciences, The University of Tokyo, 1-1-1 Yayoi, Bunkyo-ku, Tokyo 113-8657, Japan; 3 Shandong Binzhou Animal Science and Veterinary Medicine Academy, Binzhou 256600, China; wjl478@163.com; 4 Kawata Animal Clinic, 724-1 Arai, Fukaya, Saitama 366-0016, Japan; shimizu.kvc@gmail.com (H.S.); niwano0621@gmail.com (A.N.); kawata.vt@icloud.com (R.K.)

**Keywords:** bovine leukemia virus (BLV), *BoLA-DRB3* polymorphism, semen, progeny, proviral load (PVL), susceptible, resistant, inheritance, Japanese Black cattle

## Abstract

Bovine leukemia virus (BLV) is widespread globally and causes economic losses in the cattle industry. *BoLA-DRB3* is a polymorphic gene associated with the BLV proviral load (PVL), which correlates with disease progression and transmission risk. However, the distribution of *BoLA-DRB3* alleles in semen and their potential impact on the PVL of progeny remain unclear. Here, we investigated whether BLV susceptibility linked to *BoLA-DRB3* alleles in semen is inherited by progeny. We analyzed 178 commercial semen samples from Japanese Black sires and identified 20 *BoLA-DRB3* alleles and 70 genotypes. The susceptible allele DRB3*016:01 was the most frequent (26.4%), whereas resistant alleles DRB3*011:01 (5.3%) and DRB3*009:02 (0.6%) were rare. Subsequently, we collected blood samples from 200 progeny produced by artificial insemination using 36 of the 178 semen samples. Progeny derived from semen carrying at least one susceptible allele and no resistant alleles had significantly higher PVL in the blood than those derived from semen containing at least one resistant allele. These findings demonstrate that BLV susceptibility is inherited via *BoLA-DRB3* alleles in semen and highlight the potential of *BoLA-DRB3* alleles as valuable markers in breeding strategies aimed at mitigating BLV infection and transmission.

## 1. Introduction

Bovine leukemia virus (BLV) is a retrovirus linked to enzootic bovine leukosis (EBL), which is characterized by the neoplastic proliferation of B cells and can lead to lymphoma in cattle in some cases [1]. The virus integrates into the host genome as a provirus and causes lifelong infection. BLV is transmitted through both horizontal and vertical routes. Horizontal transmission includes spread through blood-sucking insects and contact with wounds or mucous membranes [2,3]. Invasive procedures that may cause bleeding, such as castration, ear tagging, dehorning, rectal examination, and blood transfusion from infected cattle, can substantially facilitate the spread of BLV [4,5]. BLV could be transmitted vertically from the dam to the progeny via the placenta, colostrum, or milk [6,7,8,9,10,11,12,13]. Although some studies have suggested that semen is a potential source of vertical transmission [14,15], the associated risk of BLV infection remains controversial [16,17,18,19,20].

Despite its eradication in parts of Western Europe [21,22], BLV remains prevalent in many regions worldwide [23]. BLV epidemics have been identified worldwide owing to improved detection using antibody- and PCR-based methods [24,25,26,27,28]. In North America, 40.0% of cattle in the United States [29,30], 39.0% in Canada [31], and 58.7% in Mexico [32] are BLV-positive. In Asia, the reported rates are approximately 10.0% in China based on a nationwide meta-analysis [33], 39.8–47.7% in Korea [34], and 28.7–40.9% in Japan [35]. Southeast Asian countries also show variable prevalence rates, such as 5.3–100.0% in Thailand [36], 4.8–9.7% in the Philippines [37], and 37.0% in Myanmar [38]. Lower rates have been reported in Mongolia (3.9%) [39], whereas African regions, such as Egypt, show moderate levels (21.5–28.0%) [40]. In addition, because BLV causes chronic infection and effective vaccines or treatments are unavailable [41], it poses a serious threat to the dairy and beef industries. Its endemic presence has substantial economic consequences, including reduced milk production [42,43,44], increased premature culling and carcass condemnation rates [45,46,47,48,49], elevated veterinary and management costs related to immunosuppression [50,51], and trade restrictions [52,53,54]. BLV infection in the United States results in an estimated annual economic loss of USD 525 million from milk loss alone [42,55,56]. In Canada, the annual economic burden per infected cow is approximately CAD 635, with losses attributed to production impacts, such as carcass condemnation owing to EBL and other factors [57]. In Japan, BLV infection in culled dairy cows with high proviral loads (PVLs) resulted in reduced carcass weight, leading to an estimated economic loss of approximately USD 1.39 million in 2017 [58].

The major histocompatibility complex (MHC) plays a crucial role in immune regulation by presenting intracellular antigenic peptides to T lymphocytes [59]. In cattle, the MHC system is referred to as the bovine leukocyte antigen (BoLA), located on chromosome 23, and is known for its high polymorphism [60]. The *BoLA-DRB3* locus has been studied among BoLA class II genes because of its significant allelic diversity and functional importance [60]. Over 386 *BoLA-DRB3* alleles are registered in the Immuno Polymorphism Database (IPD)-MHC database (https://www.ebi.ac.uk/ipd/mhc/group/BoLA/) (accessed on 9 April 2025), highlighting its potential as a genetic marker for disease resistance studies. *BoLA-DRB3* polymorphisms are associated with genetic resistance or susceptibility to various infectious diseases in cattle, including mastitis [61,62], tick-borne diseases [63], foot-and-mouth disease [64], and bovine herpesvirus 1 [65]. Recent studies have increasingly focused on the relationship between *BoLA-DRB3* polymorphisms and BLV infection outcomes. Multiple reports have demonstrated that specific *BoLA-DRB3* alleles are associated with various infection-related factors, including PVL, infectivity, persistent lymphocytosis, lymphoma development, and in utero transmission [9,66,67,68,69,70,71,72]. PVL is a major diagnostic index for determining disease progression and transmission risk [8,66,68,69,73,74,75,76,77,78,79]. Therefore, *BoLA-DRB3*’s resistant alleles associated with low BLV PVL and susceptible alleles associated with high PVL have been identified in Japanese Black and Holstein cattle [65,70,80,81]. In a large-scale study across four farms in Japan, an integrated BLV eradication program using resistant cattle as a biological barrier and preferentially eliminating susceptible cattle was established as an effective strategy to maximally reduce BLV prevalence and PVL, even in group-housed BLV-infected and -uninfected cattle in stall barns. Consequently, both BLV prevalence and mean PVL decreased on all four farms, with one farm achieving a BLV-free status [82]. This field effectiveness has been confirmed in studies in Argentina, where cattle with resistant alleles and low PVL disrupted the BLV transmission chain [74]. Furthermore, a kinetic study of the herd level of BLV infectivity in susceptible and resistant cattle in Japan from 2017 to 2019 demonstrated that the order of BLV infectivity intensity was susceptible cattle > neutral cattle > resistant cattle [83]. Moreover, a field study involving 120 dam–calf pairs across five farms in Japan revealed that the risk of vertical transmission was lower in pairs carrying *BoLA-DRB3*’s resistant alleles than in those with susceptible alleles [9]. These findings demonstrate the practicality and value of incorporating *BoLA-DRB3* genotyping into herd-level disease-control programs. Therefore, the diversity and distribution of *BoLA-DRB3* in different cattle breeds and geographic locations aid cattle breeders and veterinary geneticists in designing breeding strategies to increase the number of disease-resistant sires. This has been analyzed in various breeds worldwide [84].

The selection of sires during breeding is crucial. Since 1972, a two-stage selection has been implemented to improve carcass characteristics in Japanese Black cattle [85]. This system involves selecting bulls with satisfactory outcomes in performance tests, followed by evaluation through progeny tests. Therefore, sire selection is vital in the development of BLV-resistant herds of cattle. In addition, despite growing evidence linking *BoLA-DRB3* polymorphism to BLV infection and transmission risk, it remains unclear whether the progeny conceived via artificial insemination (AI) using semen from resistant or susceptible sires inherit these resistance or susceptibility traits. In addition, whether these traits influence their PVL remains unknown. Thus, to demonstrate whether specific *BoLA-DRB3* allele profiles in bull semen are associated with PVL in their progeny, we aimed to investigate *BoLA-DRB3* polymorphisms in 178 commercial frozen semen samples from Japanese Black sires and their progeny. We focused on the inheritance of resistant phenotypes and their potential influence on PVL. Hence, this study provides scientific evidence for genetic selection strategies to control and eradicate BLV.

## 2. Materials and Methods

### 2.1. Sample Collection, DNA Extraction, and Plasma Isolation

A total of 178 commercial frozen semen samples from top-ranking Japanese Black sires were purchased from the AI Center of the Kawata Animal Clinic (Fukaya, Japan) in an annual market survey between 2000 and 2022. These widely used semen samples were carefully selected from among the semen collected from prefectural livestock breeding laboratories and suppliers in Japan. The non-random selection process was chosen to reflect the broad distribution of commercially available semen in Japan. Whole blood samples were collected from 200 progeny born via AI using 36 selected semen samples (3 resistant, 15 susceptible, and 18 neutral sires). The 36 bulls that produced progeny for analysis were initially selected to include resistant, neutral, and susceptible sires. The bulls were further selected based on the criteria that the progeny were alive and available for blood collection with the farmer’s permission.

The semen samples were sealed in 0.5 mL polyethylene straws. Genomic DNA was extracted from 200 μL of frozen semen samples using the phenol–chloroform extraction method [86], dissolved in 30 or 90 μL Tris-ethylenediaminetetraacetic acid (EDTA) (TE) buffer, and stored at −20 °C. Based on the typical sperm concentrations in commercial bull semen, approximately 200–300 million sperm cells were present in each 200 μL sample used for DNA extraction. Blood samples were collected from the progeny of the tubes containing EDTA. For genotyping and PVL calculations, genomic DNA was extracted using the Wizard Genomic DNA Purification Kit (Promega Corporation, Tokyo, Japan), according to the manufacturer’s instructions. Peripheral blood samples were used to separate the plasma to detect anti-BLV antibodies.

### 2.2. Ethics Approval

The Animal Experiments Committee of the University of Tokyo approved this study (Approval Number: p22–2–030).

### 2.3. Detection of Anti-BLV gp51 Antibodies

Anti-BLV gp51 antibodies were detected using an anti-BLV antibody enzyme-linked immunosorbent assay (ELISA) kit (Nippon Gene, Toyama, Japan) according to the manufacturer’s instructions.

### 2.4. Determination of the BLV PVL Using BLV-CoCoMo-qPCR-2

The BLV-CoCoMo-qPCR-2 assay (Nippon Gene) was performed to determine BLV PVLs using the THUNDERBIRD Probe qPCR Mix (Toyobo, Tokyo, Japan), as previously described [27]. Briefly, the BLV long terminal region (LTR) was amplified using a degenerate BLV CoCoMo primer mix and detected using a FAM-labeled BLV MGB probe in the CoCoMoTM-BLV Primer/Probe Kit (Nippon Gene). As an internal control, *BoLA-DRA* was amplified using a DRA primer mix and detected using a FAM-labeled DRA MGB probe with a CoCoMo kit (Nippon Gene). The PCR conditions were 95 °C for 1 min, followed by 45 cycles of 95 °C for 15 s, and 60 °C for 1 min. All the amplification steps were conducted using Light Cycler^®^ 480 System II (Roche Diagnostics, Mannheim, Germany). Finally, PVL was calculated using the following formula: (number of BLV LTR copies per number of *BoLA-DRA* copies) × 10^5^. BLV PVL is expressed as the number of copies per 10^5^ cells. This method has a high sensitivity for BLV provirus detection because it detects proviruses in low-copy cows at one copy per 10^5^ cells [87]. In addition, the assay demonstrated superior sensitivity compared with those of other real-time PCR assays for molecular clones and field samples [87,88,89].

### 2.5. BoLA-DRB3 Genotyping

*BoLA-DRB3* alleles were determined using the PCR-sequence-based typing (SBT) method described by Takeshima et al. (2011) [90]. Briefly, *BoLA-DRB3* exon 2 was amplified via single-step PCR using the DRB3 forward (5′-CGCTCCTGTGAYCAGATCTATCC-3′) and reverse (5′-CACCCCCGCGCTCACC-3′) primer sets. The PCR products were purified using a FastGene Gel/PCR Extraction Kit (NIPPON Genetics Co., Ltd., Tokyo, Japan) and sequenced using FASMAC (Atsugi, Japan). Sequence data were analyzed using the Assign 400ATF ver. 1.0.2.45 software (Gonexio Genomics, Fremantle, Australia) to determine the *BoLA-DRB3* genotype. When one or two alleles were detected, the sample was considered to be homozygous or heterozygous, respectively.

Although spermatozoa are haploid cells carrying only one allele per locus, semen samples contain a heterogeneous mixture of sperm derived from both parental chromosomes. Therefore, genomic DNA extracted from whole semen reflects the diploid genotype of the individual, allowing the detection of two *BoLA-DRB3* alleles in each sample. This principle has been demonstrated in cattle by Lewin et al. (1992) [91] through single sperm typing, confirming that semen-derived DNA reliably represents the individual’s genotype, similar to somatic cell DNA.

### 2.6. Classification of BoLA-DRB3 Alleles and Genotype Grouping

Based on previously established associations between specific *BoLA-DRB3* alleles and PVL in Japanese black cattle [67,68], each allele was classified into one of three categories: susceptible, resistant, or neutral. The allele BoLA-DRB3*016:01, which has been consistently associated with elevated PVL, was classified as susceptible. The alleles BoLA-DRB3*009:02 and BoLA-DRB3*011:01, which have been associated with significantly lower PVL levels, were classified as resistant. The remaining alleles that have not shown statistically significant associations with PVL in previous studies were classified as neutral. Progeny genotypes were grouped based on the combinations of these allele categories to evaluate their association with PVL. Six genotype combinations were defined: resistant/resistant (R/R), resistant/susceptible (R/S), resistant/neutral (R/N), susceptible/neutral (S/N), neutral/neutral (N/N), and susceptible/susceptible (S/S) allele genotypes.

### 2.7. Statistical Analysis

A two-tailed Student’s *t*-test was used to compare PVL levels between the progeny carrying the susceptible and resistant *BoLA-DRB3* alleles. The equality of variances was assessed using Levene’s test. Welch’s correction was applied when equal variance was not assumed. Genotype classification was based on previously reported associations between specific *BoLA-DRB3* alleles and BLV PVL [92]. Statistical significance was set at *p* < 0.05. As only a single primary comparison was performed, no correction for multiple comparisons was applied. All the statistical analyses were performed using R version 4.4.2 with RStudio version 2024.12.1 Build 563 (Posit Software, Boston, MA, USA).

## 3. Results

### 3.1. Distribution of BoLA-DRB3 Alleles in Widely Used Commercial Frozen Semen

The distribution of *BoLA-DRB3* alleles was determined in 178 widely used commercial frozen semen samples from Japanese Black cattle using PCR-SBT targeting exon 2 of the *BoLA-DRB3* gene (Table 1). A total of 20 alleles were identified, which were reported in the IPD-MHC Database, which lists 386 known *BoLA-DRB3* alleles. The alleles with frequencies >5% were BoLA-DRB3*002:01, *005:02, *005:03, *007:01, *010:01, *011:01, *012:01, *015:01, and *016:01. Two alleles (*001:01 and *013:02) had frequencies between 2% and 5%. Eight alleles (BoLA-DRB3*005:08, *006:01, *008:01, *009:02, *020:01:02, *027:03, *034:01, and *040:02) was present at a frequency of <2%. Of the 20 alleles detected in semen, the most frequent was BoLA-DRB3*016:01 (26.4%). The second most frequent allele was BoLA-DRB3*015:01 (13.2%). The other frequent alleles were BoLA-DRB3*010:01 (10.4%), BoLA-DRB3*002:01 (8.1%), and BoLA-DRB3*005:03 (7.0%). In contrast, several rare alleles were identified, including DRB3*006:01, *020:01:02, *034:01, and DRB3*040:02, each in only one animal (0.3%).

### 3.2. Distribution of Susceptible, Resistant, and Neutral BoLA-DRB3 Alleles in Widely Used Commercial Frozen Semen

**Figure 1:**
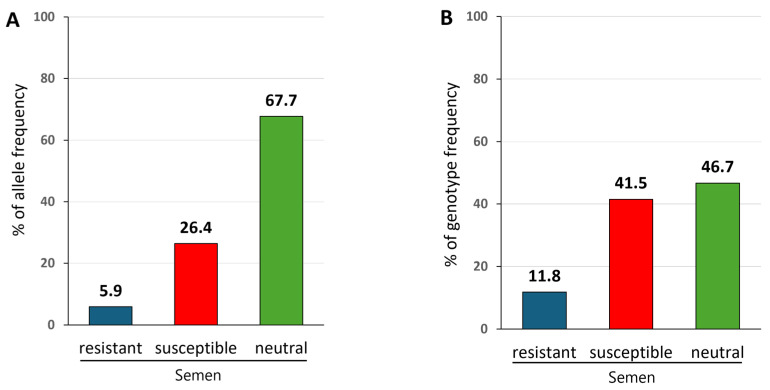
Allele and genotype frequencies of *BoLA-DRB3* in widely used commercial frozen semen from Japanese Black cattle. Genomic DNAs were obtained from 178 widely used commercial frozen semen samples from Japanese Black sires in Japan, and *BoLA-DRB3* alleles were typed using the PCR-SBT method: (**A**) Allele frequency. BoLA-DRB3*016:01 is a susceptibility-associated marker related to high PVL, BoLA-DRB3*009:02 and BoLA-DRB3*0:11:01 are resistance-associated markers related to low PVL, and all other alleles were neutral because they did not show a significant association with PVL. The X-axis shows allele classification, and the Y-axis shows the percentage of allele frequencies. (**B**) Genotype frequency. The 178 semen samples were divided into resistant, susceptible, and neutral semen groups based on the *BoLA-DRB3* Genotype: Resistant semen carried at least one resistant allele, BoLA-DRB3*009:02 or *011:01, in their genome; susceptible semen carried at least one susceptible BoLA-DRB3*016:01 without a resistant allele in their genome; and neutral semen carried other alleles in their genome. The X-axis shows semen classification, and the Y-axis shows the percentage of genotype frequencies.

Previous studies have shown that BoLA-DRB3*016:01 is associated with high BLV PVL and is, therefore, classified as a susceptible allele, whereas BoLA-DRB3*009:02 and BoLA-DRB3*0:11:01 are associated with low PVL and are considered resistant alleles in the Japanese Black breed [68]. Because none of the other alleles showed a significant association with PVL [80], they were classified as being neutral. In this study, we compared the frequencies of the *BoLA-DRB3* alleles, including the resistant and susceptible alleles. As shown in Figure 1A, 5.9% of the resistant alleles (BoLA-DRB3*009:02 and BoLA-DRB3*0:11:01), 26.4% of the susceptible alleles (BoLA-DRB3*016:01), and 67.7% of the neutral alleles were detected in 178 widely used commercial frozen semen samples. Among the resistant alleles, BoLA-DRB3*011:01 was detected in 19 samples (allele frequency = 5.3%) and BoLA-DRB3*009:02 in 2 samples (allele frequency = 0.6%) (Table 1). In contrast, the most frequent allele, BoLA-DRB3*016:01 (26.4%), was associated with susceptibility to BLV PVL (Table 1). Thus, the rate of resistant *BoLA-DRB3* alleles was lower than susceptible alleles.

### 3.3. Distribution of Susceptible, Resistant, and Neutral BoLA-DRB3 Genotypes in Widely Used Commercial Frozen Semen

We compared the frequency of *BoLA-DRB3* genotypes, including resistant and susceptible alleles, in widely used commercial frozen semen samples. Semen carrying at least one susceptible BoLA-DRB3*016:01 but no resistant allele was defined as susceptible; those carrying at least one resistant allele (BoLA-DRB3*009:02 or BoLA-DRB3*011:01) were defined as resistant; and those that did not carry susceptible or resistant alleles were defined as neutral. As presented in Table 2, 178 cattle were divided into 70 *BoLA-DRB3* genotypes among the 178 semen samples, which included 13 genotypes carrying resistant alleles, 14 carrying susceptible alleles without resistant alleles, and 43 comprising neutral alleles. As shown in Figure 1B, the results of genotype frequency showed that 11.8% resistant genotypes, 41.5% susceptible genotypes, and 46.7% neutral genotypes were detected in 178 widely used commercial frozen semen. Among the 178 semen samples, 16 (8.9%) were homozygous genotype for susceptible allele (BoLA-DRB3*016:01/*016:01), 58 (32.6%) were heterozygous genotype for susceptible and neutral allele (BoLA-DRB3*016:01/neutral), 3 (1.7%) were heterozygous for resistant and susceptible alleles (BoLA-DRB3*011:01/*016:01), 16 (8.9%) were heterozygous for resistant and neutral alleles (BoLA-DRB3*011:01/neutral), 1 (0.6%) were heterozygous for resistant and susceptible alleles (BoLA-DRB3*009:02/*016:01), 1 (0.6%) were heterozygous for resistant and neutral alleles (BoLA-DRB3*009:02/neutral), and 83 (46.7%) were homozygous for neutral alleles (neutral/neutral) (Table 3). No semen samples were homozygous for resistant alleles (BoLA-DRB3*009:02/*009:02 or BoLA-DRB3*011:01/*011:01) (Table 3). These distributions provide a foundation for further investigation of the relationship between paternal *BoLA-DRB3* genotypes and BLV resistance/susceptibility in progeny.

### 3.4. Selection of Semen and Progeny for BLV Susceptibility Evaluation

To further investigate the potential impact of the *BoLA-DRB3* genotypes of the fathers on the susceptibility or resistance of their progeny to BLV infection, we selected 36 representative semen samples from progeny subjected to an experiment with 70 *BoLA-DRB3* genotypes (Table 2). The 36 selected semen samples covered typical genotypes from the resistant (3 semen samples), susceptible (15 semen samples), and neutral (18 semen samples) categories (Table 2).

Of the progeny born by AI from the 36 selected semen samples, we selected 200 progeny from the cattle we collected peripheral blood samples (Table S1). Among the 15 selected sires carrying susceptible genotypes, 93 progeny were produced, including 51 progeny from seven sires carrying BoLA-DRB3*016:01/*016:01, 30 progeny from two sires carrying BoLA-DRB3*016:01/*005:03, and 4 progeny from two sires carrying BoLA-DRB3*016:01/*007:01 (Table 2). Among the three selected sires carrying the resistant genotypes, 41 progeny were produced: 12 from one sire carrying BoLA-DRB3*011:01/*002:01, 16 from another sire carrying BoLA-DRB3*011:01/*001:01, and 13 from a third sire carrying BoLA-DRB3*011:01/*005:02 (Table 2). Among the 18 selected sires carrying neutral genotypes, 66 progeny were obtained, including 13 progeny from 2 sires carrying BoLA-DRB3*005:03/*015:01 and 3 progeny from 2 sires carrying BoLA-DRB3*005:02/*005:03. This selection provides insights into investigating the heritability of BLV resistance and susceptibility to specific paternal *BoLA-DRB3* genotypes.

### 3.5. BLV Infection and PVL in Progeny Derived from Different Sire Genotypes

We extracted genomic DNA from peripheral blood samples of the selected 200 progeny, genotyped them to determine *BoLA-DRB3* allele polymorphisms, and performed BLV-CoCoMo-qPCR-2 to calculate the BLV PVL. Plasma was separated from the blood of cattle using ELISA to detect anti-BLV antibodies (Table S1). Of the 200 progeny, 68 (34%) tested positive for BLV provirus and anti-BLV antibodies (Table 4). Nineteen (46.3%) of the 41 progeny from resistant semen, 26 (28.0%) of the 93 progeny from susceptible semen, and 23 (34.8%) of the 66 progeny from neutral semen were BLV-positive (Table 4 and Table S1).

**Figure 2:**
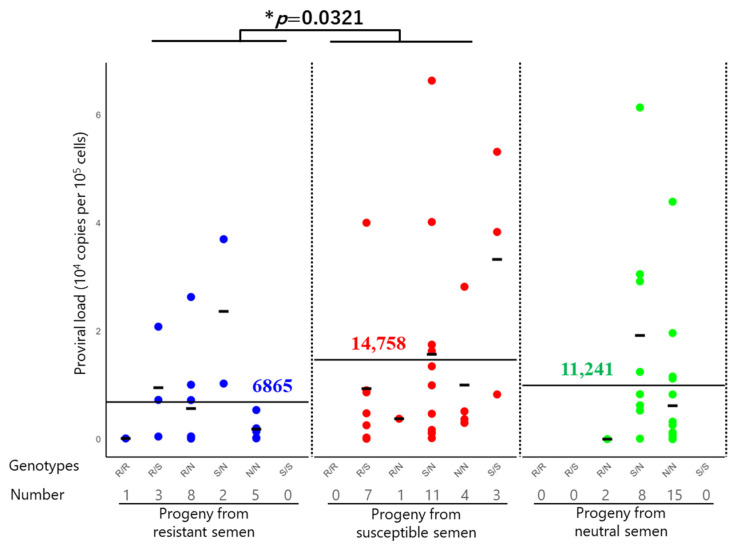
The average PVL values in the blood of the progeny derived from resistant, susceptible, and neutral semen were 6865, 14,758, and 11,241 copies per 10^5^ cells, respectively. Three resistant semen samples carried at least one resistant allele, BoLA-DRB3*009:02 or *011:01, in their genome; 15 susceptible semen samples carried at least one susceptible *allele*, BoLA-DRB3*016:01, without a resistant allele in their genome; and 18 neutral semen samples carried other alleles. Blood samples were obtained from all 200 progeny, from which DNAs were extracted. The *BoLA-DRB3* alleles were typed using the PCR-SBT method, and PVLs were measured using the CoCoMo-qPCR-2 method. Each semen group was subdivided based on the progeny genotype: resistant/resistant (R/R), resistant/susceptible (R/S), resistant/neutral (R/N), susceptible/neutral (S/N), neutral/neutral (N/N), and susceptible/susceptible (S/S) allele genotypes. The mean PVL was compared among the three groups, and the *p*-value was calculated using Student’s *t*-test after analysis of variance. Asterisks indicate significant differences (* *p* < 0.05). Only a single primary comparison was performed; thus, no correction for multiple comparisons was applied. All the statistical analyses were performed using R version 4.4.2 with RStudio (Posit Software, Boston, MA, USA).

The progeny derived from semen carrying resistant, susceptible, or neutral genotypes exhibited notable differences in the BLV PVL (Table 4 and Table S1). Figure 2 summarizes the distribution of the PVL in these groups. The average PVL in the 19 BLV-positive progeny derived from the resistant semen was 6865 copies per 10^5^ cells. Twenty-three BLV-positive progeny from neutral semen had an intermediate PVL (11,241 copies per 10^5^ cells), whereas 26 BLV-positive progeny derived from susceptible semen had the highest average PVL of 14,758 copies per 10^5^ cells. The order of the average PVL was as follows: progeny from susceptible semen > neutral progeny from neutral semen > progeny from resistant semen. Furthermore, to assess the statistical significance of PVL differences between groups, a two-tailed *t*-test following log_10_ transformation of PVL values revealed a significant difference between the resistant and susceptible semen groups (*p* = 0.032), indicating that paternal genotype may influence the PVL in the progeny (Figure 2).

The BLV-positive progeny were divided into several groups based on whether their genotypes were homozygous or heterozygous for the susceptible and resistant alleles (Figure 2 and Table 4). Further genotype-level analysis revealed that progeny carrying a homozygous genotype for resistant alleles had the lowest average PVL (124 copies per 10^5^ cells), whereas those carrying a homozygous genotype for susceptible alleles had the highest (33,725 copies per 10^5^ cells). Progeny carrying a heterozygous genotype for a susceptible allele with a resistant allele exhibited intermediate PVLs, with a mean value of 9515 copies per 10^5^ cells. Although the PVL values in this group varied across individuals, they were lower than those observed for the homozygous genotype of the susceptible allele.

## 4. Discussion

Here, we investigated the distribution of *BoLA-DRB3* polymorphisms in popular commercial semen from Japanese Black sires in Japan and the effect of paternal genotypes on PVL in their AI-derived progeny, leading to two major conclusions. First, by identifying the distribution of *BoLA-DRB3* alleles in 178 widely used commercial frozen semen samples from Japanese Black sires, we identified 20 alleles and 70 genotypes of *the BoLA-DRB3* gene and defined the distribution of susceptible, resistant, and neutral *BoLA-DRB3* alleles for BLV PVL and their genotypes. The most frequent allele, BoLA-DRB3*016:01 (26.4%), in the semen samples was susceptible to BLV PVL, whereas the frequency of the resistant *BoLA-DRB3* alleles, BoLA-DRB3*009:02 (0.6%) and BoLA-DRB3*011:01 (5.3%), was less than that of the susceptible and neutral alleles. The distribution of susceptible, resistant, and neutral alleles observed in the semen samples was consistent with that reported in previous studies based on blood samples collected from three Japanese Black cattle herds [92]. Thus, the results indicated that the distribution of resistant and susceptible alleles for BLV PVL in the blood of Japanese Black cattle was greatly affected by the frequency of *BoLA-DRB3* alleles in the semen. To the best of our knowledge, this is the first study to investigate the distribution of *BoLA-DRB3* polymorphisms in the semen of Japanese Black sires. Second, this study provides the first direct evidence that the paternal *BoLA-DRB3* genotype significantly influences BLV PVL in the progeny. This is consistent with the findings of Benitez et al. (2019), who identified breeding bulls as a potential source of BLV transmission in beef herds [16]. We collected blood from 200 progeny derived from AI using 36 of 178 semen samples and observed an association between the *BoLA-DRB3* genotype in semen and PVL in the blood samples collected from the progeny derived from their semen. The average PVL was highest in the progeny from the susceptible semen, followed by neutral, and lowest in the progeny from the resistant semen. There was a significant difference in the BLV PVL between the resistant and susceptible semen groups. This study revealed significant differences in BLV PVL in the blood of progeny derived from the susceptible and resistant semen, which concurred with the results of a previous study in which PVL in the blood was ranked in the order of susceptible, neutral, and resistant Japanese Black cattle [68]. Therefore, our investigation of the polymorphism of *BoLA-DRB3* in semen and progeny derived from semen demonstrated that paternal genotype may influence the viral burden in the progeny.

As presented in Table S2, the frequency of *BoLA-DRB3* alleles in the semen identified in this study partially aligns with that of previous studies based on blood samples from Japanese Black cattle [92]. To statistically evaluate the observed differences, we performed a chi-square test comparing the frequency of the BoLA-DRB3*1601 allele between the blood and semen samples. The results indicated no significant difference (χ^2^ = 1.91, df = 1, *p* = 0.167), suggesting that the variation is not pronounced. The most common and susceptible allele, DRB3*016:01 (allele frequency = 26.4%), was also the most frequent in blood samples (32.2%), as previously reported [92]. The frequency difference between the two sample types was within ±20%, indicating a comparable allele distribution. The population of the most frequent resistant DRB3*011:01 (allele frequency = 5.3%) in semen was like the previously reported frequency in the blood (allele frequency = 8.1%) (Table S2). These results suggest that *BoLA-DRB3* present in frozen semen is a reliable source for *BoLA-DRB3* genotyping at the population level and that the distribution status of the *BoLA-DRB3* allele in semen is a critical factor in the distribution of resistant and susceptible alleles for BLV PVL in Japanese Black cattle. Specific *BoLA-DRB3* alleles originating from common sires have been introduced into different farms of Japanese Black cattle [86]. Therefore, our principle is supported by a previous study showing that maternal genetics were somewhat fixed, implying that sire genetics were more likely to be affected in Japanese Black cattle [93]. In contrast, the difference between the current semen data and previous blood data was that the frequency of the resistant allele BoLA-DRB3*009:02 (allele frequency = 0.6% in semen) in our study was relatively low compared to that in the blood (11.1%) in the previous study (Table S2). In addition, the incidence rate of BoLA-DRB3*009:02 in Japanese Black cattle was 6.9% in Hokkaido prefecture [94], 6.8% in Miyazaki and Ooita prefecture [94], and 1.6% in Iwate [95], Japan. This allele has been identified as resistant in both Japanese Black and Holstein cattle, where it contributes to BLV control by suppressing viral replication [94]. None of the sires were homozygous for the resistant allele BoLA-DRB3*009:02, and two sires (3.2%) were heterozygous. In addition, two studies showed that all cattle carrying BoLA-DRB3*009:02 were heterozygous. In contrast, among the 178 semen samples with the susceptible allele BoLA-DRB3*016:01, a high proportion of 8.9% and 41.6% were homozygous and heterozygous, respectively. This suggests that elite AI sires may contribute to the high frequency of susceptible cattle in the Japanese Black cattle herd, resulting in an increased risk of horizontal and vertical transmission via cattle, which reduces the PVL of BLV and the development of BLV-induced lymphoma. Such allele frequency patterns in semen likely reflect past selection pressure for production traits and AI practices that involve using a few genetically similar sires. This distribution underscores the historical selection focus on production traits over disease resistance; therefore, future breeding strategies should incorporate disease susceptibility to prevent the spread of BLV infection. Notably, breeding programs may introduce selection bias in semen allele distributions, potentially influencing these patterns.

This study revealed a significant association between semen genotype and PVL in the progeny. We first collected blood from 200 progeny derived from AI, using 36 of 178 semen samples carrying 70 *BoLA-DRB3* genotypes: three sires with at least one resistant allele, 15 sires with one susceptible allele without a resistant allele, and 18 sires with a neutral allele without a susceptible or resistant allele. Furthermore, *BoLA-DRB3* typing revealed that 56% of the progeny from three resistant semen samples carried at least one resistant allele, whereas 83% of the progeny from 15 susceptible semen samples carried at least one susceptible allele. Finally, as estimated using the BLV-CoCoMo-qPCR-2 method, the average PVL was 6865 copies per 10^5^ cells in progeny from resistant semen, 11,241 copies per 10^5^ cells in progeny from neutral semen, and 14,758 copies per 10^5^ cells in progeny from susceptible semen. The average PVL in the progeny was highest in susceptible semen, followed by neutral and resistant semen. These data are consistent with previous reports that the average PVL of blood from susceptible cows is higher than resistant cows [68]. Our results indicate that disease susceptibility to BLV in semen affects BLV PVL control in the progeny. Similarly to other breeds besides the Japanese Black cattle, several results from Holstein cattle support our semen results. For example, Bai et al. (2021) [83] showed that the mean PVL of blood from 49 resistant cattle was 4216 copies per 10^5^ cells and that of 62 susceptible cattle was 19,206 copies per 10^5^ cells, indicating that the PVLs of blood from susceptible cattle were significantly higher than those from resistant cattle. In addition, an evaluation of whether the *BoLA-DRB3* polymorphism affected PVL in milk from BLV-infected dams [69] showed the same trend: the level of BLV PVL was significantly higher in milk from susceptible dams than in milk from resistant dams. Similarly to our previous study showing that *BoLA-DRB3* alleles control PVL, the current study demonstrated that progeny regulate PVL based on the *BoLA-DRB3* alleles of sires, providing evidence that resistance to BLV is inherited from sires.

Further division of susceptible and resistant progeny based on homozygous or heterozygous genotypes for susceptible or resistant alleles revealed that progeny with homozygous susceptible genotypes had the highest average PVL in blood (mean: 33,725 copies per 10^5^ cells), whereas progeny possessing homozygous resistant genotypes had the lowest average PVL in blood (mean: 124 copies per 10^5^ cells). A previous study reported that the BLV PVL in the blood or milk of cattle with homozygous susceptible genotypes was significantly higher than that in cattle with heterozygous resistant and neutral genotypes [9,68,69]. In addition, intermediate PVL levels in the blood (mean: 9362–9515 copies per 10^5^ cells) were observed in progeny that were heterozygous for resistant and susceptible genotypes, suggesting a dose-dependent effect and supporting the hypothesis that resistance is partially dominant [68]. These findings were statistically significant (*p* = 0.032) and provide strong evidence of the paternal immunogenetic influence on BLV susceptibility in the progeny. However, these results were observational, and further studies are warranted to confirm causality.

Our results are consistent with previous findings, highlighting the role of *BoLA-DRB3* polymorphisms as key genetic factors affecting BLV PVL and transmission risk [96]. For example, Borjigin et al. (2021) [9] demonstrated that Holstein dams carrying resistant alleles (DRB3*009:02, *014:01:01) had significantly lower PVLs and perinatal transmission rates than those with susceptible genotypes (DRB3*015:01, *012:01). All calves carrying resistant alleles in that study remained BLV-negative, even when born to infected dams. Although the aforementioned studies focused on maternal genetic contributions, the current findings extend this understanding by demonstrating that paternal *BoLA-DRB3* alleles also influence PVL in calves. The consistent dose–response trend observed in both studies (resistant/resistant < resistant/susceptible < susceptible/susceptible) further reinforced the immunogenic role of *BoLA-DRB3* in controlling BLV.

Although a low frequency of resistant *BoLA-DRB3* alleles may appear, this is likely to reflect a historical emphasis on economically important traits, such as growth rate, carcass quality, and reproductive efficiency in breeding programs. However, the high frequency of susceptibility-associated *BoLA-DRB3* alleles may be linked to the desirable production characteristics that contribute to their persistence in the population. *BoLA-DRB3* exerts positive and negative effects on dairy cattle productivity [62,97,98,99,100,101]. In addition, we previously reported that some resistant Holstein and Japanese Black cattle were less likely to develop EBL, whereas susceptible Holstein and Japanese Black cattle were more likely to develop high PVL [66,68,70] and EBL [66,67,102]. Therefore, resistant alleles should not be universally considered advantageous, and susceptible alleles should not be viewed as inherently negative. Therefore, rather than pursuing the fixation of resistant alleles, a more balanced and practical strategy is required for effective BLV control. This strategy is preferred for integrating *BoLA-DRB3* genotyping into sire selection protocols, with priority given to sires carrying resistant alleles for use in AI programs. In parallel, herd-level management interventions, such as an integrated BLV eradication program, use of resistant cattle with low PVL as a biological barrier, and preferential elimination of susceptible cattle with high PVL, particularly those homozygous for susceptible alleles [82], are recommended. This aligns with the growing international demand for resistance genotyped semen, enhancing the global competitiveness of the livestock genetics industry [74]. However, the relationship between *BoLA-DRB3* and reproductive performance has not yet been well studied. Therefore, to confirm the detailed effect of *BoLA-DRB3* on reproductive performance, future studies on the genetic background of cows with various traits are indispensable. Besides reproductive traits, to generalize the importance of *BoLA-DRB3* distribution in semen, further studies are needed to increase the number of semen and progeny samples and the variety of breeds, as well as to determine the maternal genotype.

Approximately 70% of the infected cattle remained asymptomatic. Most of the remaining infected cattle developed persistent lymphocytosis (PL), and only 1–5% developed. Thus, a notable reason why BLV causes the progression of different diseases is that EBL is a multifactorial disease caused by a complex interplay among viral, host genetic, and environmental factors. First, PVL increase or decrease may be induced by host genetic factors (except for *BoLA-DRB3*), such as altered expression of protein arginine-N-methyltransferase gene [103], DNA mismatch repair gene [104], and Syk enzyme [105], as well as tumor necrosis factor polymorphism [106,107]. Regarding viral factors, the relationships between PVL and a point mutation in the LTR of BLV [108] and a deletion mutation in the G4 gene of BLV [109] have been reported. These host and viral factors may cause changes in humoral and cellular immune responses, leading to changes in infectivity and PVL levels and possibly contributing to individual differences in disease susceptibility. Our result showed that there are individuals whose PVL levels cannot be explained by their *BoLA-DRB3* genotype alone. For example, the two progeny with the R/R genotype had PVL levels of 0 and 124 copies/10^5^ cells, respectively, indicating excessively low levels. However, one progeny with the R/N genotype showed a PVL exceeding 10,000 copies/10^5^ cells, and several N/N animals also exhibited relatively high PVL values. These results suggest that host and viral factors, in addition to *BoLA-DRB3* genotypes, have an impact individually or in combination. A thorough analysis of the aforementioned phenomena that occur at low external temperatures may reveal the control mechanism of PVL. In addition to viral and host factors, significant environmental factors influence BLV infection rates [35,75,110]. The environmental factors include age, herd size, herd antibody positivity rate, herd EBL incidence rate, herd PVL value, husbandry methods, housing condition, castration, summer fly infestation status, maternal infection status, and colostrum feeding from cows to calves. Furthermore, the alleles of resistance, susceptibility, and neutrality showed no correlations in progeny and infection rates (Table 2). Therefore, future studies that standardize environmental factors are warranted to clarify the influence of *BoLA-DRB3* polymorphisms on infection rates in progeny. Notably, although the blood samples from progeny were collected from nine prefectures across Japan—enhancing the geographical representativeness of the data—the maternal genotype was unavailable and could not be analyzed, which may contribute to PVL variation. Moreover, this study provides observational evidence of an association between sire genotype and progeny PVL; however, causality cannot be definitively established without further controlled studies.

Our study highlights that the susceptibility of the *BoLA-DRB3* gene to BLV PVL is inherited, as paternal genetics influences calf PVL. Furthermore, our previous study showed that the presence of susceptible or resistant alleles in cattle determines their susceptibility to BLV, regardless of whether the allele is inherited from the sire or dam [66,68,70]. However, the present study did not consider the effects of PVL on the dam. Therefore, further studies are required to investigate the influence of *BoLA-DRB3* alleles in dams and sires on BLV disease susceptibility in their calves. Although increasing BLV resistance in sire herds is a long-term goal, it can be achieved by combining genotypic screening with herd management practices. These findings provide a scientific foundation for sustainable integration of genetics into national BLV control strategies. In addition, we found that BoLA-DRB3*016:01, an allele associated with susceptibility to BLV, was most frequently observed in semen samples collected from Japanese Black cattle. This finding suggests that Japanese sires have not been selected based on resistance to BLV but rather on other significant economic traits, such as meat texture. To develop a BLV-resistant cattle herd, it is essential to promote the introduction of *BoLA-DRB3* alleles associated with BLV resistance while concurrently selecting for other desirable economic traits.

## Figures and Tables

**Table 1:** Distribution of *BoLA-DRB3* alleles in 178 commercial frozen semen samples.

*BoLA-DRB3* Allele	Allele Frequency		Susceptibility of Alleles ^b^
	n ^a^	%	
*001:01	7	(2.0)	N
*002:01	29	(8.1)	N
*005:02	22	(6.2)	N
*005:03	25	(7.0)	N
*005:08	2	(0.6)	N
*006:01	1	(0.3)	N
*007:01	19	(5.3)	N
*008:01	6	(1.7)	N
*009:02	2	(0.6)	R
*010:01	37	(10.4)	N
*011:01	19	(5.3)	R
*012:01	18	(5.1)	N
*013:02	13	(3.7)	N
*014:01:01	8	(2.2)	N
*015:01	47	(13.2)	N
*016:01	94	(26.4)	S
*020:01:02	1	(0.3)	N
*027:03	4	(1.1)	N
*034:01	1	(0.3)	N
*0 *40* *:0* *2*	1	(0.3)	N

^a^ n = number of alleles. ^b^ S = BoLA-DRB3*016:01 is associated with high BLV PVL and is classified as a susceptible allele; R = BoLA-DRB3*009:02 and BoLA-DRB3*011:01 are associated with low PVL and are classified as resistant alleles; and N = all other alleles not showing significant association with PVL are neither susceptible nor resistant alleles, and are classified as neutral alleles.

**Table 2:** Frequency of 70 *BoLA-DRB3* genotypes among 178 widely used commercial semen samples, number of selected semen samples used for progeny production, and number of progeny derived from the selected samples.

Semen				Progeny from Selected Semen	Semen				Progeny from Selected Semen
*BoLA-DRB3* Genotype	Frequency		Selected Semen	n	*BoLA-DRB3* Genotype	Frequency		Selected Semen	n
	n ^a^	%				n	%		
Resistant					*010:01/*013:02	3	1.7	0	0
*011:01/*015:01	4	2.2	0	0	*002:01/*002:01	2	1.1	0	0
*011:01/*002:01	3	1.7	1	12	*002:01/*005:02	2	1.1	0	0
*011:01/*016:01	3	1.7	0	0	*002:01/*010:01	2	1.1	0	0
*011:01/*013:02	2	1.1	0	0	*002:01/*012:01	2	1.1	0	0
*009:02/*012:01	1	0.6	0	0	*005:03/*005:03	2	1.1	1	14
*009:02/*016:01	1	0.6	0	0	*005:03/*010:01	2	1.1	0	0
*011:01/*001:01	1	0.6	1	16	*007:01/*007:01	2	1.1	0	0
*011:01/*005:02	1	0.6	1	13	*007:01/*015:01	2	1.1	2	12
*011:01/*006:01	1	0.6	0	0	*008:01/*008:01	2	1.1	0	0
*011:01/*007:01	1	0.6	0	0	*010:01/*010:01	2	1.1	0	0
*011:01/*010:01	1	0.6	0	0	*012:01/*012:01	2	1.1	1	1
*011:01/*012:01	1	0.6	0	0	*012:01/*015:01	2	1.1	1	1
*011:01/*014:01:01	1	0.6	0	0	*013:02/*015:01	2	1.1	1	1
Total	21	11.8	3	41	*015:01/*027:03	2	1.1	1	2
Susceptible					*001:01/*005:02	1	0.6	0	0
*016:01/*016:01	16	9.0	7	51	*001:01/*007:01	1	0.6	0	0
*016:01/*010:01	12	6.7	0	0	*001:01/*010:01	1	0.6	0	0
*016:01/*015:01	11	6.2	1	1	*001:01/*012:01	1	0.6	0	0
*016:01/*005:03	7	3.9	2	30	*001:01/*015:01	1	0.6	0	0
*016:01/*007:01	7	3.9	2	4	*002:01/*005:03	1	0.6	1	4
*016:01/*002:01	4	2.2	0	0	*002:01/*013:02	1	0.6	0	0
*016:01/*005:02	4	2.2	2	5	*002:01/*014:01:01	1	0.6	0	0
*016:01/*013:02	3	1.7	0	0	*005:02/*005:02	1	0.6	0	0
*016:01/*014:01:01	3	1.7	0	0	*005:02/*007:01	1	0.6	1	7
*016:01/*008:01	2	1.1	1	2	*005:02/*014:01:01	1	0.6	0	0
*016:01/*012:01	2	1.1	0	0	*005:02/*015:01	1	0.6	0	0
*016:01/*001:01	1	0.6	0	0	*005:03/*005:08	1	0.6	1	3
*016:01/*020:01:02	1	0.6	0	0	*005:03/*007:01	1	0.6	0	0
*016:01/*034:01	1	0.6	0	0	*005:03/*012:01	1	0.6	0	0
Total	74	41.6	15	93	*005:08/*007:01	1	0.6	1	2
Neutral					*007:01/*027:03	1	0.6	0	0
*002:01/*015:01	8	4.5	0	0	*010:01/*014:01:01	1	0.6	1	1
*010:01/*015:01	6	3.4	1	1	*010:01/*040:02	1	0.6	0	0
*005:03/*015:01	5	2.8	2	13	*012:01/*013:02	1	0.6	0	0
*005:02/*005:03	4	2.2	2	3	*012:01/*027:03	1	0.6	0	0
*005:02/*010:01	4	2.2	1	1	*014:01:01/*015:01	1	0.6	0	0
*005:02/*012:01	3	1.7	0	0	Total	83	46.6	18	66

^a^ n = number of semen samples or number of progeny blood samples.

**Table 3:** Distribution of *BoLA-DRB3* genotypes in 178 commercial frozen semen samples from Japanese Black cattle.

Susceptibility of Semen	*BoLA-DRB3* Genotype	Frequency	
		n ^d^	(%)
Resistant ^a^	*009:02/*009:02	0	(0.0)
	*009:02/*016:01	1	(0.6)
	*009:02/neutral	1	(0.6)
	*011:01/*011:01	0	(0.0)
	*011:01/*016:01	3	(1.7)
	*011:01/neutral	16	(8.9)
	*009:02/*011:01	0	(0.0)
	Total	21	(11.8)
Susceptible ^b^	*016:01/*016:01	16	(8.9)
	*016:01/neutral	58	(32.6)
	Total	74	(41.5)
Neutral ^c^	neutral/neutral	83	(46.7)
	Total	83	(46.7)
	Total	178	(100.0)

^a^ semen samples carrying at least one resistant allele (BoLA-DRB3*009:02 or BoLA-DRB3*011:01) were defined as resistant. ^b^ semen samples that carried at least one copy of the susceptible BoLA-DRB3*016:01 but did not carry a resistant allele were defined as susceptible. ^c^ semen samples that carried neither resistant nor susceptible alleles were classified as neutral. ^d^ n = number of semen samples.

**Table 4:** *BoLA-DRB3* genotypes and BLV infection status in progeny derived from the selected semen.

Susceptibility of Selected Semen (Heads)	Progeny					
	Genotype	BLV-Infected Rate		Average PVL ^e^		PVL Range
		BLV-Positive n ^d^	Tested n (%)			
Resistant (3)	R/R ^a^	1/2	(50.0)	124		124–124
	R/S ^b^	3/5	(60.0)	9515		480–20,813
	R/N ^c^	8/16	(50.0)	5663		97–26,305
	S/N	2/6	(33.3)	23,643		10,284–37,002
	N/N	5/12	(41.7)	1835		140–5384
	Total	19/41	(46.3)	6865		
Susceptible (15)	S/S	3/13	(23.1)	33,725		8272–53,180
	S/N	11/55	(20.0)	15,745		1274–66,356
	R/S	7/9	(77.8)	9362		72–40,052
	R/N	1/1	(100.0)	3770		3770–3770
	N/N	4/15	(26.7)	10,007		3000–28,205
	Total	26/93	(28.0)	14,758		
Neutral (18)	S/N	8/22	(36.4)	19,188		83–61,382
	R/N	0/2	(0.0)	ND ^f^		ND
	N/N	15/42	(35.7)	6179		26–43,941
	Total	23/66	(34.8)	11,241		
Total		68/200	(34.0)	11,363		

^a^ R = resistant allele. ^b^ S = susceptible allele. ^c^ N = neutral allele. ^d^ n = number of progeny samples. ^e^ PVL = proviral load (Copies per 10^5^ cells). ^f^ ND = not detected.

## Data Availability

The original contributions presented in the study are included in the article; further inquiries can be directed to the corresponding author.

## Supplementary Materials

The following supporting information can be downloaded at https://www.mdpi.com/article/10.3390/pathogens14090837/s1. Table S1: *BoLA-DRB3* alleles and proviral load (PVL) in 200 progeny samples derived from semen; Table S2: Comparison of *BoLA-DRB3* allele frequency between blood and semen samples from the Japanese Black breed.
